# Clinical features and infection risks of Chinese children with different types of Gaucher disease

**DOI:** 10.3389/fped.2025.1595394

**Published:** 2025-09-05

**Authors:** Chuan Gan, Yuanyuan Wu, Tao Qin

**Affiliations:** ^1^ Department of Infectious Diseases Children’s Hospital of Chongqing Medical University, National Clinical Research Center for Child Health and Disorders, Ministry of Education Key Laboratory of Child Development and Disorders, Chongqing Key Laboratory of Child Rare Diseases in Infection and Immunity, The First Batch of Key Disciplines on Public Health in Chongqing, Chongqing, China; ^2^ Health Medicine Center, The Second Affiliated Hospital of Chongqing Medical University, Chongqing Medical University, Chongqing, China

**Keywords:** Gaucher disease, clinical feature, infection risk, Chinese children, gene mutation

## Abstract

**Background:**

Gaucher disease (GD) is a rare autosomal recessive disorder caused by mutations in the glucocerebrosidase1 (*GBA1*) gene. Reports on the clinical presentations of various types of GD in Chinese children are scarce, and there is limited research addressing co-occurrence of GD with bacterial (including tuberculosis), viral, or fungal, infections. Pediatric GD typically manifests with greater severity due to developmental vulnerability of organ systems and immature immunity, leading to heightened infection risks. Unlike non-GD children, those with GD exhibit multiorgan involvement (e.g., hepatosplenomegaly, cytopenias) that predisposes them to opportunistic infections. In this study, we describe the clinical features and infection risks associated with different types of GD in Chinese children.

**Methods:**

This study was done in Children's hospital of Chongqing Medical University. Seventeen patients aged <18 years, diagnosed with GD from January 2008 to December 2019, were enrolled. Clinical symptoms, laboratory results, mutation genotypes, and imaging data were collected for analysis.

**Results:**

Of the 17 patients, 9 were diagnosed with Type 2 GD, while 4 each had Type 1 and 3 GD. Median (interquartile range) age of onset was 7 (3.0–18.5) months. Approximately two-thirds of patients experienced malnutrition, and most exhibited hepatosplenomegaly and hematological abnormalities. Anemia was the most frequent hematological disorder, followed by thrombocytopenia, with almost half developing leukopenia. Liver function abnormalities were common, particularly in Type 2 GD, and characterized by elevated aspartate aminotransferase and glutamyl transpeptidase levels, prolonged prothrombin time, and decreased albumin. Patients with Type 2 GD had increased susceptibility to infections, with respiratory failure from severe infections a leading cause of death. Genome sequencing revealed a novel deletion mutation (c.787_c.788 delAA) in the *GBA1* gene associated with Type 2 GD.

**Conclusion:**

In pediatric patients with Gaucher disease, Type 1 GD is associated with worse hematological impairment, while Type 2 GD involves significant hepatic insufficiency and heightened susceptibility to infections.

## Impact of this article

• Clinical manifestations of Gaucher disease (GD) vary significantly.
• Almost half of pediatric patients with all types of GD develop leukopenia.
• Hepatic insufficiency is related to both the type and severity of GD in pediatric patients.
• Patients with Type 2 GD have an increased susceptibility to infections, particularly with gram-negative bacteria.

## 1 Background

Gaucher disease (GD, OMIM #230800) is a rare autosomal recessive disorder caused by mutations in the glucocerebrosidase1 (*GBA1*) gene, leading to decreased β-glucocerebrosidase (GCase) activity and consequent issues with lysosomal storage (1). The consequences of this deficiency are generally attributed to the accumulation of the GCase substrate, GlcCer, in macrophages, inducing their transformation into Gaucher cells (2). Gaucher cells subsequently infiltrate the bone marrow, spleen, liver, and multiple systemic organs, eliciting corresponding pathological changes. GD can be categorized into three subtypes based on the presence of neuronopathy and the rate of disease progression. Type 1 GD is non-neuronopathic and the most prevalent form worldwide. Type 2 GD, the acute neuronopathic form, manifests in infancy and patients with this form of GD have a life expectancy of less than 2 years. Type 3 GD is an adolescent onset form with subacute neuronopathy. However, this traditional classification is increasingly being challenged by a growing body of evidence indicating a correlation between Type 1 GD and neurological impairments, such as Parkinson's disease and peripheral neuropathies (3, 4). GD diagnosis relies on detecting reduced GCase activity or direct sequencing of the *GBA1* gene. Despite the importance of timely diagnosis and treatment, GD is often missed or misdiagnosed, due to its rarity and heterogeneous symptoms. The incidence of GD is low, with an estimated rate of 1/40,000–1/60,000 births in the general population (2, 5). Clinicians, especially generalists, are often unfamiliar with the early clinical features of GD. Additionally, the signs and symptoms of GD are highly variable, making it challenging to differentiate the disease from other conditions. In a survey by Mistry et al., when patients presented with typical features, only 20% of hematologists or oncologists considered GD in their differential diagnosis (6). Another study by Mehta et al. found that almost one in six patients remained undiagnosed for 7 years or more after initial consultation (7). Hematologists, hepatologists, and pediatricians are the main specialists to whom patients first present (8, 9). Gaucher disease patients in China experience elevated misdiagnosis incidence, substantial diagnostic delays, and financially burdensome treatment expenses (10). Although timely diagnosis can minimize the impact of misdiagnoses and avoid unnecessary invasive diagnostic procedures, as well as being crucial for optimal therapy and patient management, particularly since enzyme replacement and substrate reduction therapies have proven effective in treating certain types of GD. Due to prolonged diagnostic delays coupled with restricted access and prohibitive costs of therapies, Chinese patients often lose confidence in future treatment prospects (10).

Gaucher disease patients may exhibit functional immunodeficiency, clinically manifesting as impaired antimicrobial host defense, delayed resolution of infections, increased infection susceptibility, and increased risk of autoimmune disorders (11–13). Additionally, they demonstrate predisposition to recurrent infections (14). Zahran et al. reported that children with GD1 exhibit a significant expansion of activated T cells in peripheral blood, correlating with heightened infection risks (15). However, systematic data on infection susceptibility in Gaucher disease remain limited, and no study has yet compared infection risk across the different GD subtypes. Therefore, we analyzed Chinese pediatric patients to enhance awareness among hematologists, pediatricians, and other clinicians regarding the distinctive clinical and immunological profile of this rare disorder, with the aim of informing timely diagnosis, treatment selection, and long-term management.

## 2 Methods

### 2.1 Patients

This study included 17 patients aged <18 years, diagnosed with GD between January 2008 and December 2019 at the Children's Hospital of Chongqing Medical University. Diagnoses were based on GBA1 activity in peripheral leukocytes, Bone marrow cytology and/or gene sequencing, and were confirmed by Sanger sequencing. Exome capture was conducted using a GenCap Liquid Capture Kit (MyGenostics, MD, USA), and sequencing performed using the Illumina HiSeq 2500 platform. Average sequencing read-depth was 300×, with >90% of the exome covered at ≥20×. The study was approved by the Ethics Committee of the Children's Hospital of Chongqing Medical University [Approval No. 2025 Lunar Review (Research) No. (034)] and conducted in accordance with the Declaration of Helsinki.

### 2.2 Clinical data

Clinical symptoms, laboratory results, mutation genotypes, and imaging data were extracted from electronic medical records. GD types were classified by three pediatric specialists and follow-up was conducted through clinical visits or telephone counseling, with two patients lost to follow-up. Pneumonia was diagnosed according to the radiologists’ report, based on pulmonary infiltrates or opacities. Three or more episodes of respiratory infections within a year were defined as recurrent respiratory tract infections. Upper respiratory tract infection was defined as presenting with symptoms of upper respiratory infection and a record of visiting the outpatient department. Urinary infection was defined as symptoms of urinary irritability and/or abnormal urinalysis findings, along with a positive urine culture result.

### 2.3 Statistical analysis

Continuous variables are described using mean and standard deviation or median with interquartile range (IQR) values. Categorical variables are expressed as counts and/or percentages. One-way ANOVA in GraphPad Prism 5 was used to analyze differences between continuous variables, with *P* values ≤ 0.05 considered statistically significant.

## 3 Results

### 3.1 Demographic characteristics of patients with GD

From 2008 to 2019, 17 pediatric GD patients were enrolled: Type 1 (*n* = 4), Type 2 (*n* = 9), Type 3 (*n* = 4); Female predominance: 64.7% (11/17). Median (IQR) age of onset was 7 (3.0–18.5) months. Malnutrition affected 70.6% (12/17) of patients: 75% of those with Type 1 (3/4), 66.7% with Type 2 (6/9), and 75% with Type 3 (3/4) GD. Fifteen patients received at least one follow-up via telephone or outpatient clinic, and a high mortality rate was observed among patients with Type 2 GD. One patient with Type 1 GD died 3 years after diagnosis, while no deaths occurred in the Type 3 GD group during the follow-up period. Hepatosplenomegaly and hematological abnormalities were common; anemia (94.1%) and thrombocytopenia (82.4%) were the most frequent hematological findings, followed by leukopenia (47.1%). Patients with Type 2 GD also more frequently exhibited growth retardation relative to those with Types 1 and 3, with a tendency towards short stature (Table 1).

**Table 1:** Demographic features of Gaucher diseases in children.

Gaucher diseases	Type 1	Type 2	Type 3	Total
	(*n* = 4)	(*n* = 9)	(*n* = 4)	(*n* = 17)
Sex (%)				
Male	3 (75)	1 (11.1)	2 (50)	6 (35.3)
Female	1 (25)	8 (88.9)	2 (50)	11 (64.7)
Median age of onset (mo, IQR)	8.5 (2.5–21.5)	5.0 (2.5–7.5)	90 (8.3–156)	7.0 (3–18.5)
Nutritional status (%)				
Normal	1 (25)	3 (33.3)	1 (25)	5 (29.4)
Mild malnutrition	1 (25)	4 (44.5)	-	5 (29.4)
Moderate malnutrition	2 (50)	1 (11.1)	3 (75)	6 (35.3)
Server malnutrition	-	1 (11.1)	-	1 (5.9)
Clinical manifestation (%)				
Hepatomegaly	3 (75)	8 (88.9)	3 (75)	14 (82.4)
Splenomegaly	3 (75)	9 (100)	4 (100)	16 (94.1)
Anemia	3 (75)	9 (100)	4 (100)	16 (94.1)
Thrombocytopenia	4 (100)	6 (66.7)	4 (100)	14 (82.4)
Leukopenia	2 (50)	3 (33.3)	3 (75)	8 (47.1)
Apnea	-	5 (55.6)	-	5 (29.4)
Diahrrea	-	3 (33.3)	1 (25)	4 (23.5)
Dwarfism	2 (50)	-	3 (75)	5 (29.4)
Growth retardation	1 (25)	7 (77.8)	-	8 (47.1)
Follow up				
Survive	2 (50)	-	4 (100)	6 (35.3)
Death	1 (25)	8 (88.9)	-	9 (52.9)
Lost followup	1 (25)	1 (11.1)	-	2 (11.8)

### 3.2 Differences in laboratory findings among patients with the three types of GD

Significant differences in hematological and hepatic function were observed across GD subtypes (Table 2). Patients with Type 1 GD had severe hematological impairment: low leukocyte (3.6 ± 1.7 × 10^9^/L) and hemoglobin levels (58.3 ± 20 g/L), while those with Type 2 GD showed pronounced hepatic dysfunction: increased aspartate aminotransferase (AST; 96.6 ± 47.7 mmol/L) and gamma-glutamyl transferase (GGT; 58.4 ± 79.1 U/L), and decreased albumin (ALB; 34.9 ± 7.1 g/L). Activated partial thromboplastin time (APTT) was prolonged in all groups, but the difference did not reach significance. Prothrombin time (PT) was significantly prolonged in patients with Type 2 GD (13.7 ± 6.09 s). These observations indicate that hematological deficits dominated in Type 1, while hepatic insufficiency correlated with Type 2 severity. Moreover, patients with type 1 exhibited significantly higher levels of alkaline phosphatase (ALP) and lactate dehydrogenase (LDH) than those with types 2 and 3. This finding may reflect the fact that both enzymes are not exclusively hepatic; they are also associated with the hematopoietic and skeletal systems.

**Table 2:** The hematology and biochemistry findings in each group.

Gaucher diseases	Type 1	Type 2	Type 3	*P* value		
				GD1 vs. GD2	GD1 vs. GD3	GD2 vs. GD3
Hematology						
White blood cell (×10^9^/L)	3.6 ± 1.7 (1.4–8.2)	6.4 ± 3.2 (2.7–18)	5.8 ± 4.3 (5.2–16)	0.007*	0.014*	0.762
Hemoglobin (g/L)	58.3 ± 20 (26–115)	90.3 ± 13.9 (60–126)	94.2 ± 18.2 (55–117)	0.011*	0.003*	0.291
Platelet (×10^9^/L)	100.0 ± 40.3 (3–241)	98.0 ± 66.7 (22–481)	157.0 ± 139.0 (16–415)	0.525	0.121	0.021*
Liver function						
ALB (g/L)	42.4 ± 3.63 (33.7–48.3)	34.9 ± 7.1 (20.3–46.6)	39.4 ± 5.99 (28.9–45.7)	0.004*	0.884	0.017*
ALT (mmol/L)	50.0 ± 15.8 (11.3–90.6)	41.9 ± 21.8 (19.1–112)	28.7 ± 8.2 (17.7–41.5)	0.012*	0.001*	0.024*
AST (mmol/L)	68.4 ± 21.6 (33–122)	96.6 ± 47.7 (50.4–248)	57.4 ± 30.7 (10.2–108)	0.002*	0.967	0.001*
AKP (U/L)	222.0 ± 64.3 (101–454)	129.0 ± 79.6 (66–336)	157.0 ± 67.2 (86.4–232)	0.001*	0.009*	0.294
GGT (U/L)	22.5 ± 10.3 (10.0–64.0)	58.4 ± 79.1 (13.0–436)	27.4 ± 19.8 (1.2–55)	0.022*	0.712	0.034*
LDH (U/L)	795 ± 718 (160–1,029)	439 ± 247 (140–980)	305 ± 214 (121–576)	0.002*	0.036*	0.294
PT (s)	13.8 ± 0.91 (11.4–14.4)	13.7 ± 6.09 (10.3–35.2)	13.0 ± 1.35 (11.5–16.1)	0.028*	0.926	0.020*
APTT (s)	40.3 ± 1.54 (37.5–43.4)	39.3 ± 24.8 (23.1–126.8)	40.8 ± 8.14 (26.4–56.5)	0.964	0.927	0.294

* *P* < 0.05.

### 3.3 Inflammation and infection in patients with GD

A high infection rate (9/17, 52.9%) was observed, particularly in patients with Type 2 GD (8/9, 88.9%). Respiratory infections were most common, accounting for 77.8% (7/9) of cases. Additionally, one patient experienced a urinary tract infection. A rare case of lymphadenopathy due to *Mycobacterium bovis* following Bacillus Calmette-Guerin (BCG) vaccination was detected in one patient (Table 3). Bacterial infections were predominant, particularly gram-negative bacteria, including *E. coli*, *K. pneumoniae*, *A. baumannii*, and *Moraxella catarrhalis*. One patient had combined infections with adenovirus, parainfluenza virus, and *C. albicans*. Overall, patients with Type 2GD had an increased susceptibility to infections.

**Table 3:** Clinical manifestations and infection profiles in Gaucher's disease patients.

Patient	Type	Age of onset (month)	First clinical manifestation at our hospital	Site of infection at visit to our hospital	Pathogen	History of lower respiratory tract infection	History of upper respiratory tract infection	Antimicrobial
1	2	3	Hepatomegaly, splenomegaly, axillary lymphadenopathy	Lymphadenopathy	Mycobacterium bovis	On three occasions, diagnosed with pneumonia	Two episodes	Isoniazid (ivgtt, 8d),
								Rifampin (ivgtt, 8d),
								Ethambutol (oral, 8d)
4	2	1	Hepatomegaly, splenomegaly, developmental delay	Urinary	Escherichia coli	On two occasions, diagnosed with pneumonia	Two episodes	Ceftriaxone (ivgtt, 7d)
5	2	7	Anemia, splenomegaly	Respiratory	Escherichia coli, Staphylococcus aureus	—	—	Meropenem (ivgtt, 4d)
8	2	4	Hepatomegaly, splenomegaly	Respiratory	Staphylococcus aureus	On two occasions, diagnosed with Severe pneumonia	One episodes	Latamoxef (ivgtt, 2d), Flucloxacillin (ivgtt, 6d), Azithromycin (oral, 3d)
9	1	13	Hepatomegaly, splenomegaly, anemia	Respiratory	Moraxella catarrhalis	On five occasions, diagnosed with pneumonia	None	Azithromycin (ivgtt, 7d), Piperacillin and Tazobactam (ivgtt, 4d), Amoxicillin and Clavulanate Potassium (ivgtt, 3d)
11	2	11.5	Developmental delay	Respiratory	Adenovirus, Parainfluenza virus, Candida albicans	On one occasions, diagnosed with Severe pneumonia	Four episodes	Ceftizoxime (ivgtt, 9d), Piperacillin and Tazobactam (ivgtt, 21d)
12	2	8	Convulsion	Respiratory	Klebsiella pneumoniae, Acinetobacter baumannii	—	—	Ceftizoxime (ivgtt, 18d), Piperacillin and Tazobactam (ivgtt, 20d)
15	1	4	Thrombocytopenia	Respiratory	—	On two occasions, diagnosed with pneumonia	Two episodes	Piperacillin and Tazobactam (ivgtt, 3d)
16	2	7	Convulsion	Respiratory	Escherichia coli	On one occasions, diagnosed with pneumonia	Two episodes	Ceftizoxime (ivgtt, 25d), Piperacillin and Tazobactam (ivgtt, 15d)

### 3.4 Immunoglobulins and lymphocyte subsets

Five patients underwent immunoglobulin testing, with no significant abnormalities observed in IgG, IgM, and IgE in any of them. One patient had an increase in IgA, while another patient had a decrease in this immunoglobulin. Additionally, four patients underwent lymphocyte subtype analysis, of which two had elevated double-negative T cells (Table 4).

**Table 4:** Immunoglobulin, lymphocyte subsets for the patient in the study.

Patient	Type	IgA (n.v.0.1–1.29) g/L	IgG (n.v.2.86–16.8) g/L	IgM (n.v.0.21–1.92) g/L	IgE (n.v. <150 IU/ml)	CD3+ cells (% total lymphocytes) (n.v.50–84)	CD4+ cells (% total lymphocytes) (n.v.27–51)	CD8+ cells (% total lymphocytes) (n.v.15–44)	DNT-cells (% total lymphocytes) (n.v. <1.8)	CD19+ cells (% total lymphocytes) (n.v.5–18)	CD56+16+ cells (% total lymphocytes) (n.v.7–40)	CD4\CD8 (n.v.0.7–2.8)
1	2	0.992	10.9	1.64	8.4	52.68	25.28(L)	25.58	1.82(H)	23.5(H)	22.6	0.99
3	3	—	—	—	—	66	33	27	6(H)	10	23	1.24
4	2	0.08(L)	4.62	0.756	2.83	—	—	—	—	—	—	—
5	2	0.292	5.21	0.626	1	85(H)	60(H)	24	1	9	4(L)	2.5
6	3	1.35(H)	8.34	0.667	2.1	—	—	—	—	—	—	—
11	2	0.122	5.45	0.442	2.6	63.6	46.35	16.54	0.71	33.12(H)	3.14(L)	2.8

H, higher than normal range; L, lower than normal range; NK, natural killer cells; DNT, double-negative T cell; n.v., normal value.

### 3.5 Bone marrow cytology examination results

Bone marrow cytology examinations were completed for 12 patients: two with Type 1, seven with Type 2, and three with Type 3 GD. The granulocyte series/erythroid series ratio was lower in all patients with Type 1 and higher in all those with Type 2 GD, except for one patient with Type 2 GD, who exhibited an inversion. One patient each with Type 1 and Type 2 GD had abnormal granulocyte morphology, while no abnormalities were observed in patients with Type 3 disease; however, all three patients with Type 3 GD exhibited abnormal erythroid morphology, suggesting that this may be a common finding (Table 5). A significant reduction in platelets was observed in two patients, one with Type 1 and the other with Type 2 GD; no platelet abnormalities were detected in patients with Type 3 disease.

**Table 5:** Bone marrow cytology in GD patients.

Patient	Type	Granulocyte series/Erythroid series	Granulocyte (%)	Erythroid (%)	Lymphocyte (%)	Immature lymphocytes (%)	Granulocyte morphology	Erythroid morphology	Lymphocyte morphology	Platelet status	Bone marrow examination report
10	1	1.2:1	42.5	36.5	21	0	Normal	Normal	Normal	Rare	Gauchers cells were found, conformed with Gaucher's disease
14	1	0.4:1	21	55	23	2.5	Abnormal	Abnormal	Normal	Normal	Gauchers cells were found, conformed with Gaucher's disease
4	2	1.7:1	30.5	18	49	4	Normal	Normal	Normal	Normal	Gauchers cells were found, conformed with Gaucher's disease
5	2	2.7:1	60	22	16.5	2.5	Normal	Abnormal	Normal	Normal	Gauchers cells were found, conformed with Gaucher's disease
8	2	1.9:1	45.5	24.5	29.5	3.5	Normal	Normal	Normal	Normal	Gauchers cells were found, conformed with Gaucher's disease
11	2	4.7:1	49	10.5	36.5	3	Normal	Normal	Normal	Normal	Gauchers cells were found, conformed with Gaucher's disease
12	2	3.9:1	65.5	17	13.5	1.5	Normal	Normal	Normal	Normal	Gauchers cells were found, conformed with Gaucher's disease
13	2	0.6:1	21.5	34.5	32	3	Normal	Normal	Normal	Normal	Gauchers cells were found, conformed with Gaucher's disease
16	2	1.5:1	47	31.5	21.5	1.5	Abnormal	Normal	Normal	Rare	Gauchers cells were found, conformed with Gaucher's disease
2	3	1.30:1	39.5	30.5	29	0	Normal	Abnormal	Normal	Normal	Gauchers cells were found, conformed with Gaucher's disease
3	3	4.56:1	61.5	13.5	22	0	Normal	Abnormal	Normal	Normal	Gauchers cells were found, conformed with Gaucher's disease
6	3	1.3:1	47.5	37.5	14.5	1	Normal	Abnormal	Normal	Normal	Gauchers cells were found, conformed with Gaucher's disease

### 3.6 Imaging characteristics of patients with GD

**Figure 1:**
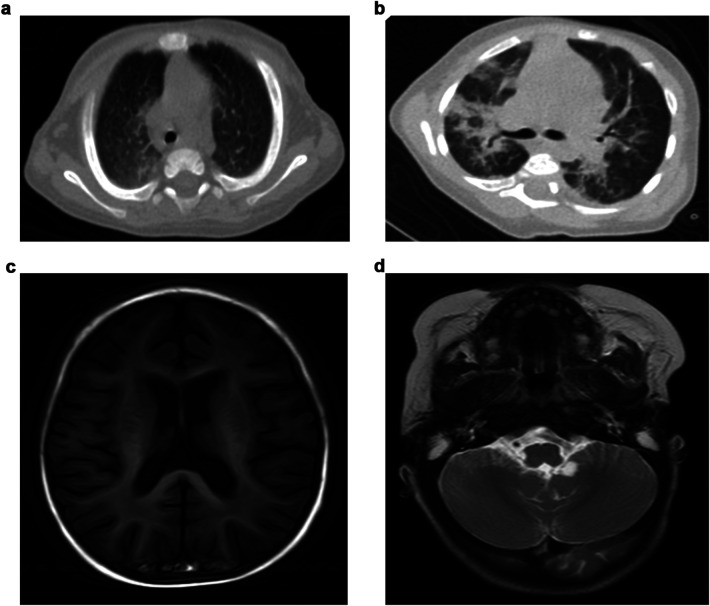
Imaging characteristics of GD patients.

Abdominal ultrasonography indicated hepatosplenomegaly without liver fibrosis. Chest computed tomography (CT) scans in eight patients revealed abnormalities in six of them, with the most frequent being consolidated patches in bilateral lung lobes. One patient exhibited multiple patch shadows in the left lobe, with bilateral pleural effusions (Figures 1a,b). Cranial magnetic resonance imaging (MRI) revealed encephalatrophy in five of six patients, with two having arachnoid cysts (Figures 1c,d); whether or not arachnoid cysts are a complication of GD requires further investigation.

### 3.7 Genotype and prognosis of patients with GD

Gene sequencing of samples from eight patients revealed the c.1448T > C (p.L483P) mutation was common (6/8). Other mutations detected included c.475C > T (p.R159W), c.501-1G > C, c.1255G > A (p.D419N), c.787_c.788delAA, c.689T > G (p.V230G), c.460G > A (p.G154R), and c.1081G > C (p.D361H) (Table 6). A novel *GBA1* mutation genotype (c.787–788 delAA, p.L263V fs*9; c.1448T > C, p.L483P) was identified in a patient who initially presented with neurological disorders at an early stage; motor development retardation onset occurred at 2 months, followed by a rapid disease progression that culminated in the patient's death within only 6 months.

**Table 6:** Genotype of GBA gene in GD patients.

Case	Type	Mutation	Mutation Type
C9	1	c.1448T > C (p.L483P)	Homozygous
C14	1	c.1448T > C(p.L483P)	Compound heterozygous
		c.475C > T(p.R159W)	
		c.680A > G,681T > G(p.N227N/K/S/R)	
		c.721G > A (p.G241R)	
C1	2	c.1448T > C (p.L483P)	Compound heterozygous
		c.501-1G > C (splicing)	
C8	2	c.1448T > C(p.L483P)	Homozygous
C12	2	c.1255G > A(p.D419N)	Compound heterozygous
		c.1448T > C(p.L483P)	
C13	2	c.787_c.788 delAA (p.L263V fs*9)	Compound heterozygous
		c.1448T > C(p.L483P)	
C16	2	c.689T > G(P.V230G)	Homozygous
C7	3	c.460G > A(p.G154R)	Compound heterozygous
		c.1081G > C(p.D361H)	

None of the patients had received enzyme replacement therapy or substrate reduction therapy. Nine of fifteen patients died during the follow-up period. All patients in the Type 2 GD group succumbed within 6 months, due to rapid progression of neuropathy and/or respiratory failure from infection. One patient in the Type 1 GD group died 3 years after disease onset, due to respiratory and heart failure. High mortality was observed, particularly in patients with Type 2 GD. Patients with Type 3 GD exhibited chronic progression during regular follow-up.

## 4 Discussion

Our study presents a comprehensive characterization of Chinese children with GD, detailing their clinical manifestations, biochemical markers, immunophenotypes, genotypes, and systemic organ involvement, together with systematic longitudinal follow-up of disease progression and prognosis. Type 1 GD patients exhibited pronounced hematologic impairment, whereas type 2 patients showed marked hepatic injury. For the first time, we demonstrate subtype-specific differences in infection risk among Chinese pediatric cohorts, revealing a significantly higher susceptibility and risk in type 2 patients. These findings underscore the importance of tailored infection surveillance and management strategies, particularly for individuals with type 2 GD.

The hematological manifestations of GD include splenomegaly, thrombocytopenia, anemia, and cytopenia; these disorders are attributable to hypersplenism, bone marrow infiltration by Gaucher cells, or intrinsic hematopoietic cell deficiencies (16–18). Occasionally, the phenomenon of erythrophagocytosis has been reported in GD (19, 20), suggesting immune dysfunction in the context of hematological abnormalities. Kaplan et al. found that anemia and severe hepatosplenomegaly were more common in young children, while thrombocytopenia was prevalent in patients up to 18 years old. Type 1 GD is associated with more severe hematological impairment than that detected in other GD types (21). Patients with Type 2 GD exhibit similar findings, with anemia and thrombocytopenia appearing later and/or with moderate severity (22). In the current study, patients in the Type 1 GD group exhibited worse hematological impairment than those in the other groups. In one case, Type 1 GD was comorbid with beta-thalassemia, and the patient presented with severe anemia, which could have interfered with appropriate interpretation of anemia in Type 1 GD. Almost half of patients in this study exhibited leukopenia, which is generally an uncommon hematological impairment. Long-term monitoring of a larger patient cohort is necessary to better evaluate the hematological characteristics of patients with GD.

Neurological impairment is common in Types 2 and 3 GD and can include brainstem degeneration, fine motor dysfunction, hypertonia, rigidity, and seizures (23, 24). Ophthalmological abnormalities, such as horizontal saccade and strabismus, are among the earliest central nervous system features in patients with GD, followed by hypertonia, rigidity, opisthotonus, swallowing impairment, and seizures (25, 26). Apnea caused by laryngeal spasms is a symptom of late stage of Type 2 GD and contributes to shortened life expectancy (27). In our study, neuronopathy was more severe in patients with Type 2 GD than in the Type 3 GD group. Symptoms including psychomotor retardation, seizures, and strabismus were observed in both groups, while hypertonia/hypotonia, opisthotonos, and laryngeal spasms were characteristic signs in the Type 2 GD group (data not shown).

Recent research has confirmed that neuronopathy also occurs in the non-neuronopathic form of GD (i.e., Type 1 GD); In a study, microstructural alterations of brain white matter were discovered in children with Type 1 GD using diffusion tensor magnetic resonance imaging (28), and the researchers compared the topological properties of the brain network in patients with Type 1 GD with those of healthy controls. Children with GD showed abnormal small-world topology and altered distribution of motor- and sensory-related regions throughout the course of the disease (29). These findings support the notion that GD should be considered a continuum, rather than as a set of discrete categories (8).

Bone impairment in GD includes osteopenia, osteonecrosis, bone crises, chronic bone pain, pathological fractures, lytic lesions, and skeletal deformities, which are prevalent symptoms of Types 1 and 3 GD (30–32). Kaplan et al. reported that approximately 80% of pediatric patients had at least one radiological skeletal abnormality at diagnosis (21). Erlenmeyer flask deformity (49%) and bone marrow infiltration (38%) were the two most common radiological manifestations, with older children more prone to severe skeletal problems. In another report, 63% of patients with GD aged <10 years presented with Erlenmeyer flask deformity (33). Importantly, children with GD who manifest bone pain are often incorrectly characterized as having growing pains (34).

In our cohort, the cytological findings from bone marrow examinations exhibited significant differences across the various subtypes of GD. Patients with type 1 consistently demonstrated a uniformly low myeloid-to-erythroid (M/E) ratio, while all type 2 patients presented with an elevated M/E ratio. Additionally, type 3 patients consistently displayed abnormal erythrocyte morphology. The potential correlation between these cellular aberrations and distinct skeletal manifestations warrants further investigation.

Hepatic involvement in GD includes hepatomegaly, liver fibrosis, and hepatocellular carcinoma. In this study, we found that liver dysfunction severity correlated with GD type, with significant abnormalities detected in patients with Type 2 GD (35–37). Patlas et al. analyzed the clinical features of 103 children with GD and found that all exhibited hepatomegaly (38). Serai et al. evaluated liver stiffness in patients with Type 1 GD using magnetic resonance elastography and discovered a significant correlation between liver stiffness and disease severity (39). Starosta et al. investigated hepatic impairment in 42 patients with GD, finding that the rates of liver enzyme abnormalities, hepatomegaly, and steatosis were 68%, 67%, and 8%, respectively; six patients underwent liver biopsy, among which three had liver fibrosis and two had steatohepatitis; one patient developed hepatocellular carcinoma (40). Several studies have observed prolongation of PT and APTT in both adults and children (41–43). In the current study, the severity of liver dysfunction was related to disease type. Patients with Type 2 GD exhibited elevated AST and GGT, but a decrease in ALB. Further, both PT and APTT were prolonged in all three groups, with significantly elevated APTT in the Type 2 GD group, indicating inferior liver function. Our data indicate that liver dysfunction severity correlates with GD type, with Type 2 GD associated with significant abnormalities.

In GD, lung lesions are caused either by Gaucher cell infiltration, leading to interstitial lung disease, including pulmonary fibrosis, pulmonary arterial hypertension, restrictive ventilatory impairment, and intrapulmonary arterial-venous shunts, or by repetitive aspiration pneumopathies, which lead to chronic lung disease (22, 44). Pulmonary involvement is severe in patients with Type 2 GD, often presenting as recurrent respiratory infections. Similarly, most patients with Type 2 GD in our study reported bacterial respiratory infections. These findings align with current evidence indicating that GD can confer increased susceptibility to infection and a propensity for recurrent infectious episodes (14). The heightened infection risk in GD may be attributable to dysfunction of immune cells, including monocytes, macrophages, dendritic cells, T cells, and B cells, as well involvement of cytokines, such as MCP1/CCL2, CXCL8/IL8, CXCL1, IL-1β, IFNγ, and TNFα, are implicated in GD progression (45–50). Gene-knockout mice exhibit impaired thymic maturation (51, 52) and a loss of macrophage function (53). An Egyptian pediatric study reported an increased in CD8⁺ T lymphocytes alongside a reduction in NK cells in GD patients (54). Although these alterations in immune cells and mediators may contribute to heightened susceptibility, the relationship between subtype-specific immunological profiles and differential infection risk in GD remains to be elucidated.

Patients with GD may present with hypergammaglobulinemia. Studies have reported elevated IgG, IgM, and IgA levels in GD patients, with IgG elevation being the most persistent and pronounced. Following enzyme replacement therapy, IgG levels typically rise further, whereas IgA and IgM usually normalize or decrease (55–58). Regrettably, no patients in our cohort received ERT and no significant immunoglobulin elevation was observed. These findings suggest that immunoglobulin dysregulation may be uncommon among Chinese pediatric GD patients. However, given the limited sample size, larger prospective studies with systematic immunoglobulin profiling are warranted to clarify the true prevalence of immunoglobulin abnormalities in this population.

GD may exhibit autoimmune lymphoproliferative syndrome (ALPS)-like characteristics and defects in FAS-mediated apoptosis. Although double-negative T cells are generally considered specific to ALPS, elevated levels of double-negative T cells have also been observed in patients with GD. In our study, lymphocyte subset analysis was performed on samples from four patients, of which 2 (50%) had elevated levels of double-negative T cells, consistent with a report by Maurizio Miano and colleagues (59). This finding also suggests an immunophenotypic overlap between GD and ALPS, indicating that genetic diagnosis is the gold standard for differentiation between these conditions. Over 400 *GBA1* mutations have been identified, with some linked to specific phenotypes and ethnic groups (60, 61). The N370S mutation is highly prevalent in the Ashkenazi Jewish and Caucasian populations and is considered “neuroprotective,” as patients with this mutation, in both homozygous and heterozygous states, are less likely to develop neurological impairment (34, 62). The L444P mutation is common in Asian countries and associated with neurological impairment (63–66). Further, patients with Type 3 GD homozygous for the D409H mutation present with characteristic heart valve damage (67–69). Overall, although GD diagnosis depends on *GBA1* mutation, the relationships between genotypes and phenotypes appear to be weak.

ERT has significantly improved most manifestations of GD1 and has enhanced the quality of life for patients (70). Additionally, it may provide benefits for patients with GD3 who experience chronic visceral involvement (8). However, pediatric Gaucher disease remains under-recognized in China, with only a small percentage of affected children receiving ERT. Therefore, systematic collection and analysis of long-term clinical data from a larger cohort is essential to facilitate early recognition and accurate diagnosis, enabling timely initiation of ERT and ultimately improving patient outcomes.

## 5 Conclusion

In Chinese children with Gaucher disease, Type 1 GD may be associated with more pronounced hematological impairment, whereas Type 2 GD is potentially characterized by significant liver damage and heightened susceptibility to infections. Timely diagnosis and appropriate management are crucial for improving the quality of life in pediatric patients with GD in China. Given the clinical and immunophenotypic overlap between GD and IEI, genetic diagnosis is the gold standard.

## Data Availability

The datasets presented in this study can be found in online repositories. The names of the repository/repositories and accession number(s) can be found in the article/Supplementary Material.
